# Perinatal Exposure to Low Doses of Dioxin Can Permanently Impair Human Semen Quality

**DOI:** 10.1289/ehp.1002134

**Published:** 2011-01-24

**Authors:** Paolo Mocarelli, Pier Mario Gerthoux, Larry L. Needham, Donald G. Patterson, Giuseppe Limonta, Rosanna Falbo, Stefano Signorini, Maria Bertona, Carla Crespi, Cecilia Sarto, Paul K. Scott, Wayman E. Turner, Paolo Brambilla

**Affiliations:** 1 University Department of Laboratory Medicine, Hospital of Desio, Monza Brianza, Italy; 2 University Milano-Bicocca, School of Medicine, Milano, Italy; 3 Division of Laboratory Sciences, National Center for Environmental Health, Centers for Disease Control and Prevention, Atlanta, Georgia, USA; 4 EnviroSolutions Consulting, Inc., Jasper, Georgia, USA; 5 ChemRisk Inc., Pittsburgh, Pennsylvania, USA

**Keywords:** breast-feeding, dioxin, environmental endocrine disrupters, human sperm impairment, human sperm quality, perinatal exposure, reproductive hormones, TCDD

## Abstract

**Background:**

In recent decades, young men in some industrialized areas have reportedly experienced a decrease in semen quality.

**Objective:**

We examined effects of perinatal dioxin exposure on sperm quality and reproductive hormones.

**Methods:**

We investigated sperm quality and hormone concentrations in 39 sons (mean age, 22.5 years) born between 1977 and 1984 to mothers exposed to dioxin after the accident in Seveso, Italy (1976), and 58 comparisons (mean age, 24.6 years) born to mothers exposed only to background dioxin. Maternal dioxin levels at conception were extrapolated from the concentrations measured in 1976 serum samples.

**Results:**

The 21 breast-fed sons whose exposed mothers had a median serum dioxin concentration as low as 19 ppt at conception had lower sperm concentration (36.3 vs. 86.3 million/mL; *p* = 0.002), total count (116.9 vs. 231.1; *p* = 0.02), progressive motility (35.8 vs. 44.2%; *p* = 0.03), and total motile count (38.7 vs. 98 million; *p* = 0.01) than did the 36 breast-fed comparisons. The 18 formula-fed exposed and the 22 formula-fed and 36 breast-fed comparisons (maternal dioxin background 10 ppt at conception) had no sperm-related differences. Follicle-stimulating hormone was higher in the breast-fed exposed group than in the breast-fed comparisons (4.1 vs. 2.63 IU/L; *p* = 0.03) or the formula-fed exposed (4.1 vs. 2.6 IU/L; *p* = 0.04), and inhibin B was lower (breast-fed exposed group, 70.2; breast-fed comparisons, 101.8 pg/mL, *p* = 0.01; formula-fed exposed, 99.9 pg/mL, *p* = 0.02).

**Conclusions:**

*In utero* and lactational exposure of children to relatively low dioxin doses can permanently reduce sperm quality.

For the past 50 years, scientists have debated the issue of a reported decline in human semen quality, especially in Western countries (Carlsen et al. 1992; Swan et al. 2000). There is now a general consensus that human sperm quality has declined over time in different areas of the world (Auger et al. 1995; Jørgensen et al. 2001; Swan et al. 2003), especially in younger men (López-Teijòn et al. 2008; Zheng et al. 1997), with indications of concurrent male sub- or infertility (Bonde et al. 1998; Paasch et al. 2008; Zheng et al. 1997).

No clear causes have been identified; however, selected widespread persistent environmental endocrine-disrupting chemicals, such as 2,3,7,8-tetrachlorodibenzo-*p*-dioxin (TCDD) and other dioxin-like chemicals, are suspected to be potential etiologic agents, as demonstrated in experimental animals, but no definitive data are available for men.

Recently, we showed that exposure in infancy to low doses of dioxin induces a sperm concentration reduction of about 25%; however, no reduction is observed if similar exposures occur during adulthood (Mocarelli et al. 2008). These data are consistent with animal models, which have demonstrated that the most sensitive times for these effects were exposures while *in utero* and during breast-feeding (Faqui et al. 1998).

In infants, after 4–5 months of breast-feeding, dioxin serum concentrations increase up to 2-fold (Abraham et al. 1998). In that period, there is a hormonal surge (Forest et al. 1973; Quigley 2002), defined as “neonatal minipuberty,” in which an increase of follicle-stimulating hormone (FSH) and inhibin B (Andersson et al. 1998) induces and reflects a strong proliferation of Sertoli cells (Cortes et al. 1987), the number of which determines how many germ cells can develop into spermatozoa (Sharpe et al. 2003). In addition to exposure during *in utero* development, exposure to TCDD within this developmental window could induce a direct or indirect toxic effect (i.e., imbalance of the hormonal equilibrium), impairing such proliferation and reducing sperm production in adults.

In this study, we assessed the association between human dioxin exposure *in utero* and during breast-feeding and adverse human adult male reproductive outcomes, as measured in men whose mothers were exposed to dioxin as a result of the trichlorophenol plant explosion near Seveso, Italy, in July 1976 (Mocarelli et al. 1991; Needham et al. 1997–1998).

## Materials and Methods

### Participants

All 78 men, 18–26 years old, born between March 1977 and January 1984 to 73 Caucasian women who lived in the most dioxin-polluted areas near Seveso, Italy, in 1976 (Eskenazi et al. 2004; Mocarelli et al. 1991; Needham et al. 1997–1998) were invited to participate. Thirty-nine of these men, who are the sons of 36 of the 73 women (mean age, 24.8 ± 5.6 years at the date of accident), actually participated; 21 were breast-fed, and 18 were formula-fed (Table 1). This further subdivision allowed us to separate the exposed men into two groups: men who had been exposed *in utero* only (those formula-fed and not breast-fed), and men who had been exposed perinatally—that is, both *in utero* and during nursing (breast-fed) (Table 1).

The exposure status of the mothers of the exposed men (either participating or refusing) was verified by measuring dioxin levels in their serum, which was collected soon after the explosion. There were no significant differences in median TCDD serum concentrations in mothers of refusing men compared with the mothers of participating ones (36 vs. 51.7 ppt). Twenty exposed mothers breast-fed 21 sons, and 17 exposed mothers formula-fed 18 sons. Of these mothers, three each had two sons: One mother had a son in 1977 who was formula-fed, and another in 1981 who was breast-fed; one mother had a son in 1978 and another in 1980, both of whom were breast-fed; and one mother had a son in 1977 and another in 1983, both of whom were formula-fed. Sons of dioxin-exposed women whose spouses were also exposed, as well as all men with reported diseases (Table 1), were excluded.

As a control, a comparison group of 123 consecutive healthy volunteer permanent blood donors (in Italy, blood donors have medical examinations performed every 3 months) of the same age and similar socioeconomic status as the exposed men, but whose mothers had not lived in the TCDD-contaminated areas, were invited to participate; they lacked additional accidental dioxin exposure (but did have the background exposure), and they were comparable in terms of breast-feeding and formula feeding. Fifty-eight of these men actually participated; 36 were breast-fed, and 22 were formula-fed (Table 1).

All participants completed a questionnaire on health, socioeconomic status, smoking and drinking habits, and working conditions. Participants also provided information on their weight at birth and how long they nursed as infants. They were screened for hidden diseases by clinical laboratory tests (aspartate aminotransferase, alanine aminotransferase, γ-glutamyl transferase, total cholesterol, high-density lipoprotein cholesterol, low-density lipoprotein cholesterol, C-reactive protein, glucose, creatinine, complete blood cell count and differential, hemoglobin, hepatitis B surface antigen, hepatitis B core antibody, hepatitis C, urine analysis) and donated blood and semen samples.

The study protocol was approved by institutional human subjects committee (Istituto di Ricovero e Cura a Carattere Scientifico Don Gnocchi, Milano, Italy). All study participants gave written informed consent.

### Laboratory data

Semen samples were collected from participants as a postmasturbatory at-home semen sample, which was transported to the Desio Hospital laboratory at about 37°C and kept at that temperature until examination, which occurred within 1 hr from ejaculation. Blind tests were performed by the same two technicians according to the World Health Organization (WHO 1999) recommendations. Sperm percentages of A + B grades of motility, that is, progressive motility, were assessed at 400× magnification on a microscope heating stage (37°C) in duplicate, and concentration was measured using a Bürker-Türk chamber at phase contrast (400×). Morphology was evaluated by the same observer on 300 Papanicolaou-stained sperm per slide (David et al. 1975; Jouannet et al. 1988).

For serum hormone analysis, fasting blood samples were obtained on the same morning as the semen collection. An aliquot of serum was stored frozen at −80°C, and hormone levels were measured in large batches in order to reduce interassay variability. FSH, inhibin B, serum 17-β-estradiol (E_2_), and luteinizing hormone (LH) were measured according to established immunometric methods. Testosterone was measured by radioimmunoassay. Quality control protocols were strictly applied for all tests using Westgard’s multirule (Westgard QC 2009). Intra- and interassay coefficients of variation were, respectively, 2.7% and 4.5% for FSH, 4.2% and 6.7% for inhibin B, 2.5% and 4.6% for E_2_, 1.8% and 3.4% for LH, and 3.4% and 6.6% for testosterone. Sensitivity was 0.1 IU/L for FSH, 7.0 pg/mL for inhibin B, 15 pg/mL for E_2_, 0.05 mIU/mL for LH, and 0.02 ng/mL for testosterone.

All serum TCDD measurements taken from maternal serum samples stored frozen since 1976–1977 were conducted by isotope-dilution high-resolution mass spectrometry at the Centers for Disease Control and Prevention (Atlanta, GA, USA) (Patterson et al. 1987). The estimated individual maternal serum TCDD concentration at the time of conception was calculated for 20–42 years of age by using the TCDD serum concentration in 1976–1977 and the TCDD half-life for women of that age, estimated to be an average of 4 years (range, 2.1–6.7 years) (Kreuzer et al. 1997). For the equation and an example of calculation, see Supplemental Material (doi:10.1289/ehp.1002134). Serum samples collected in 2002–2003, both from the case men and from their comparisons, were also analyzed for dioxin.

### Statistical analysis

A general database was established and maintained using SAS software (version 8.2; SAS Institute Inc., Cary, NC, USA). Sperm and hormone data were fitted with a general linear model that included group, lactation class, and group × lactation class interaction as terms. Covariates included age at time of tests, length of abstinence in days, smoking habits (the total number of cigarettes smoked per day during months of habitual smoking), chemical exposures (mostly organic solvents, adhesives, paints, colors, and powders), body mass index (BMI) at the moment of the study, alcohol use (grams per day), educational level, and employment status. Scale transformations were applied to approximate normal distribution and homoscedasticity. Sperm concentration, total sperm count, progressive motile sperm count, and E_2_, testosterone, and FSH concentrations were log transformed, whereas semen volume and LH and inhibin B concentrations were square root transformed. Results were expressed as back transformation of least squares means (i.e., the means adjusted for all the terms in the model). Two types of comparisons were made: among groups within lactation class, and among lactation classes within group.

## Results

### Dioxin exposure

In 1976, the median serum TCDD concentrations in women whose eligible sons did and did not participate in the study were similar (51.7 vs. 36 ppt). We assumed the serum TCDD concentrations in the comparison group mothers at conception (Table 2) were 10 ppt in 1976–1977 (Eskenazi et al. 2004). All mothers at the time were also exposed to a background of dioxin-like chemicals [polychlorinated dibenzofurans (PCDFs) and some polychlorinated biphenyls (PCBs)], constituting a total of about 90 ppt of dioxin toxic equivalent (TEQ) concentration, which is added to the concentration of TCDD (Eskenazi et al. 2004).

Median maternal TCDD concentrations (Table 2) measured in serum collected in 1976 and extrapolated to conception levels for mothers who breast-fed or formula-fed their children were 19.0 and 27.9 ppt, respectively, and were not statistically different. Serum TCDD concentrations in sons after 4–5 months of breast-feeding are considered 2-fold those of the mothers (Abraham et al. 1998).

Serum TCDD concentrations measured at the time of this study in the exposed breast- and formula-fed groups (average, 2.4 ppt and 1.1 ppt, respectively) and their respective comparison groups (average, 1.8 ppt and 1.0 ppt, respectively) were not statistically different.

### Semen quality

**Figure 1:**
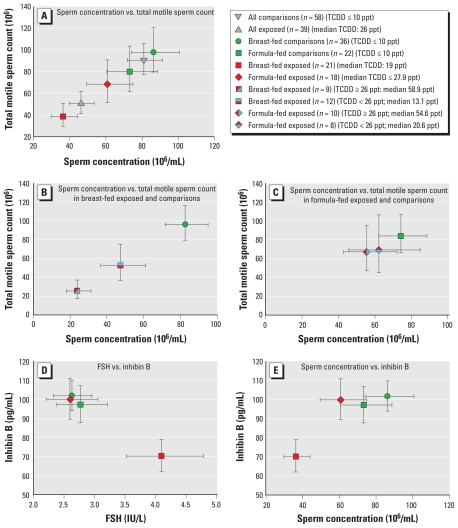
Differences in sperm variables, FSH, and inhibin B between *in utero* and breast- or formula-fed men exposed to dioxin and comparisons. (*A*) Lower sperm concentration and the lower total motility of all TCDD-exposed men (median maternal TCDD at conception, 26 ppt) with respect to comparisons was related mainly to breast-feeding (see also Table 3). (*B*) The nine breast-fed exposed men belonging to the two highest quartiles (median maternal TCDD at conception, 58.9 ppt) had both lower median sperm concentration (23.8 × 10^6^/mL) and lower motility (*p* = 0.0003, *p* = 0.003, respectively) than did the 36 breast-fed comparisons (82.5 × 10^6^/mL). Also, the 12 exposed breast-fed men belonging to the two lowest quartiles (median maternal TCDD at conception, 13.1 ppt) had lower (*p* = 0.05) sperm concentration (47.3 × 10^6^/mL) than did the 36 breast-fed comparisons (82.5 × 10^6^/mL). (*C*) Sperm variables did not statistically differ among all the formula-fed men exposed to more or less than 26 ppt of TCDD and the formula-fed comparisons. (*D*) The effect of dioxin through breast-feeding on FSH (increasing) and inhibin B (decreasing) values, compared with exposed formula-fed and comparison men, was significant and permanent. (*E*) The low sperm concentration of TCDD in exposed breast-fed men was strongly correlated to low inhibin B concentration (a marker of Sertoli cells). The formula-fed exposed men and comparisons had normal inhibin B concentration.

About 50% of both the exposed and comparison group men recruited for this study complied with semen examination (Table 1). Figure 1 and Table 3 list variables for exposed men and their comparisons. The 39 men exposed *in utero* and during nursing had significantly decreased sperm concentration (*p* = 0.01), total sperm count (*p* = 0.03), and number of total motile sperm (*p* = 0.05) relative to the 58 men in the comparison group (Table 3, Figure 1A).

A stronger significant effect appeared (Table 3) when comparing the 21 breast-fed exposed sons and the 36 breast-fed comparisons (sperm concentration, *p* = 0.002; total sperm count, *p* = 0.02; total motile sperm count, *p* = 0.01; progressive motility, *p* = 0.03; see Figure 1A). In contrast, we observed no statistical differences between the 18 formula-fed exposed men and the 22 formula-fed comparisons or between the 36 breast-fed and 22 formula-fed comparisons (Table 3). On the other hand, we observed an almost significant (*p* = 0.07) sperm concentration decrease in the 21 breast-fed exposed men compared with the 18 formula-fed exposed men. Figure 1A summarizes these observations, with TCDD values.

In examining any differences in outcome, we found that the nine breast-fed men belonging to the two highest quartiles of TCDD serum concentrations (median TCDD, 58.9 ppt) did show lower sperm concentration (*p* = 0.0003) and total motility (*p* = 0.003) than the 36 breast-fed comparisons (Figure 1B); in the equivalent formula-fed groups, we observed no statistical differences between the 10 exposed men (median TCDD, 54.6 ppt) and the 22 comparisons (Figure 1C). We observed similar effects for total sperm motility (Figure 1B,C). For the men in the two lowest quartiles, who had serum TCDD concentrations < 26 ppt (median TCDD, 13.1 ppt), the only difference was a lower sperm concentration (*p* = 0.05) in the 12 exposed breast-fed men with respect to the 36 comparison breast-fed men (Figure 1B).

These data also showed that a much lower semen concentration was present in the breast-fed men with the highest exposure, but the dose–response model did not reach statistical significance; we observed only a tendency, possibly because of the small number of cases. We did not observe this result with the formula-fed exposed men.

We observed no statistically significant difference for sperm morphology parameters between exposed and comparison groups.

### Hormones

The 21 breast-fed exposed men had higher FSH concentrations than did both the 36 breast-fed comparison men (*p* = 0.03) and the 18 formula-fed exposed men (*p* = 0.04; Table 3), whereas inhibin B was significantly decreased (*p* = 0.01 and *p* = 0.02, respectively). Figure 1D,E, illustrates the relationships between inhibin B and FSH, and inhibin B and sperm concentration in the different conditions.

We observed no statistically significant differences for E_2_, testosterone, and LH between exposed and comparison groups.

## Discussion

The results of this study clearly indicate that continuous exposure of males starting from low concentrations of dioxin before and after birth due to the mother being exposed during the Seveso accident, and then due to breast-feeding during “neonatal minipuberty,” results in a permanent impairment of the reproductive system (reduction of about 50% of sperm concentration and total sperm count and about 20% of sperm progressive motility, and increase of FSH with decrease of inhibin B) in young adulthood.

This impairment is not seen in males who were born to similarly exposed Seveso mothers who did not breast-feed. In fact, this group of men had sperm counts similar to those of the breast-fed and formula-fed comparison group.

The breast-fed exposed men had lower sperm variables than did the breast-fed comparison and formula-fed exposed men. In the latter case, statistical significance was not reached, probably because of the small number of cases. These observations on semen quality are considerably reinforced by corresponding changes in hormone levels: decreased inhibin B and increased FSH found in the breast-fed exposed group.

We had the rather unique opportunity to discriminate TCDD exposure with respect to background levels. Men exposed to rather high maternal serum TCDD concentrations at birth, such as 54.6 ppt (the 75th percentile of formula-fed exposed children), plus the then background value of 90 ppt of dioxin-like chemicals, did not show differences compared with the breast- and formula-fed comparison group members.

At the age of 4–5 months, formula-fed infants almost double their birth weight and halve the dioxin concentration, to about 27 ppt for the formula-fed exposed group and 5 ppt for the formula-fed comparison group, whereas the breast-fed comparison group approximately doubles the concentration to 20 ppt because of background dioxin exposure. On the other hand, the median of 19 ppt of dioxin in the breast-fed exposed group increases with breast-feeding to about 40 ppt. Our data show that impaired semen quality in adulthood results from exposure *in utero*, as well as continuous exposure through breast-feeding during “neonatal minipuberty,” with adverse effects starting with a dioxin exposure range of 19–40 ppt and higher (the concentrations of background dioxin-like chemicals must also be added). Similar doses of TCDD administered to rhesus monkeys during pregnancy and lactation have recently been shown to produce lower semen quality in their young adult offspring (Arima et al. 2009). It would be interesting to investigate the effect of TCDD administration only during lactation.

Our observations show for the first time that continuous exposure of the developing human male *in utero*, and even more so during lactation, the “neonatal minipuberty period,” to modest elevations of dioxin above background exposure levels can permanently impair later adult sperm production.

In fact, the only similar data (Guo et al. 2000) are related to *in utero* and nursing exposure of children to high doses of burned PCBs/PCDFs, but not to TCDD. That study demonstrated a reduction in sperm motility, but not density, as well as reduced egg penetration ability. Unfortunately, data concerning the specific concentration of the toxicants in maternal blood at conception were not available.

### Mechanism of action

Dioxin and the structurally related PCDF chemicals mediate their toxic effect (but with different potencies) mainly as a ligand to the same aryl hydrocarbon receptor (AHR)/AHR nuclear translocator (ARNT) complex that binds to the xenobiotic responsive element (XRE; also called dioxin-responsive element, DRE) on target DNA. The widely expressed AHR/ARNT, when ligand activated, interacts with several transcription factors, steroids, and growth factors and possibly plays a critical constitutive role, especially in early tissue development. Through targeted gene networks, AHR/ARNT regulates or directly intervenes in reproductive system development (Schultz et al. 2003).

As our study shows, the untimely interference of dioxin *in utero* and within the first 4–5 months of life (the “neonatal minipuberty”) (Forest et al. 1973; Quigley 2002) results in a permanent impairing effect. It is during this time that the human testes almost double their volume (Cortes et al. 1987), due in great part to Sertoli cell proliferation, which determines spermatogenic potential (Sharpe et al. 2003). An indication of possible Sertoli cell number (or functionality) reduction is given by the decrease of inhibin B, a marker of their activity, associated with the decrease of sperm concentration, not only in the exposed breast-fed with respect to the comparison breast-fed group, but also in the exposed breast-fed compared with the exposed formula-fed group. The increase of FSH associated with the decrease of inhibin B (as observed here) has been reported to be a very sensitive marker for impaired spermatogenesis in men (von Eckardstein et al. 1999).

It is well established that sons born to mothers who smoked heavily in pregnancy have a 20–50% reduction in sperm concentration in adulthood (Jensen et al. 2005; Jensen et al. 2004; Ramlau-Hansen et al. 2007), reduced blood levels of inhibin B, and increased FSH (Storgaard et al. 2003), compared with unexposed males. No information is provided about smoking during breast-feeding in these studies, but it is likely that exposure to the chemicals in tobacco smoke occurred both prenatally and postnatally, similar to our dioxin study. This is relevant to our data because a common mechanism of the phenomenon we observed and the one due to smoking could be the action on XRE (Kasai et al. 2006) of some polycyclic aromatic hydrocarbon (PAH) compounds contained in cigarette smoke, using the same AHR as dioxin.

It has also been shown that PAHs have a negative impact on rat and human Sertoli cell function (Coutts et al. 2007; Raychoudhury and Kubinski 2003).

### Relevance to general population and individuals

These observed effects of perinatal dioxin exposure on lowering adult sperm counts might also explain, in part, the wider semen quality reduction reported in young men in some industrialized regions, such as in Spain, Denmark, and Germany (Andersen et al. 2000; López-Teijòn et al. 2008; Paasch et al. 2008; Zheng et al. 1997). The men observed in these studies were in fact born in the 1970s and 1980s to mothers born in the 1950s and 1960s, a period when environmental concentrations of dioxin and dioxin-like chemicals peaked (Hagenmaier and Walczok 1996). The bioaccumulation of these compounds with age is well demonstrated, and a concurrent effect of maternal smoking could also contribute to this phenomenon.

Because of environmental policies instituted throughout most of Europe and the United States, TCDD and total TEQ concentrations in women 20–40 years of age have recently decreased sharply, to less than 2 ppt and 12 ppt, respectively (Päpke 1998; Patterson et al. 2008). Thus, current serum dioxin levels in infants in these areas, even after being increased 2- to 3-fold because of breast-feeding, are far from the concentrations that would yield adverse effects.

## Conclusions

Our data indicate for the first time that environmental chemical exposure to dioxin continuously throughout the perinatal period permanently reduces semen quality and sperm counts in young men and demonstrates that the male reproductive system is dramatically sensitive to the action of dioxin, starting from rather low doses (~ 19 ppt) *in utero* and doubled by breast-feeding during “neonatal minipuberty,” resulting in permanent impairment in the adult (reduction of ~ 50% of sperm concentration and total sperm count and ~ 20% of sperm progressive motility, with increased FSH and decreased inhibin B).

Although our findings are derived from examining a unique cohort of men exposed perinatally to supranormal dioxin levels, they may have relevance to the wider population of young men in industrialized countries and may partly explain the reported widespread occurrence of low sperm counts in young men in Europe. Our findings also raise concern for those areas of the world in which rapid industrial development may cause widespread distribution of environmental endocrine-disrupting chemicals, such as dioxin and dioxin-like chemicals.

Furthermore, our data should encourage studies of semen quality in men of appropriate ages, comparing breast-fed and formula-fed groups, especially if this could be linked to proximity to polluted areas at the time of perinatal life.

## Figures and Tables

**Table 1:** Characteristics of study participants according to dioxin exposure of the mother and to the type of nursing.

Characteristic	Exposed group	Comparison group
Recruited for the study (*n*)	78	123
Refused (*n*)	37	62
Interested (*n*)	41	61
Compliance (%)	53	50
Excluded from the study (*n*)^a^		
Varicocele	1	2
Varicocele and cryptorchidism	1	1
Participants (*n*)	39	58
Age [years (mean ± SD)]	22.5 ± 2.2	24.6 ± 2.0
Age of mothers at conception [years (mean ± SD)]	28.2 ± 5.4	28.1 ± 4.8
Nursing		
Breast-fed [*n* (%)]	21 (54)	36 (62)
Duration of breast-feeding [months (mean)]^b^	4.06	5.03
Formula-fed [*n* (%)]	18 (46)	22 (38)
BMI (kg/m^2^) [*n* (%)]		
< 25	33 (85)	48 (83)
25–30	6 (15)	10 (17)
> 30	—	—
Days of abstinence^c^ (mean ± SD)		
All	4.2 ± 1.57	4.2 ± 1.77
Breast-fed	3.9 ± 1.58	4.4 ± 1.79
Formula-fed	4.5 ± 1.56	4.0 ± 1.77

a Excluded for self-reported causes (questionnaire) or because of pathologic results of clinical laboratory tests (see “Materials and Methods”).

b Data on duration of breast-feeding were missing for four comparisons.

c Days without ejaculation before semen sampling.

**Table 2:** Serum TCDD concentrations (percentile distribution) of mothers at exposure in 1976 and extrapolated values at conception of their sons.

	Percentile (ppt)^a^				
Mothers	5th	25th	Median	75th	95th
At exposure (July 1976)					
All mothers (*n* = 36)	17.0	26.6	51.7	115.0	321.0
Mothers who breast-fed (*n* = 20)	17.0	25.2	46.8	115.0	321.0
Mothers who formula-fed (*n* = 17)	19.0	29.1	55.7	87.6	301.0
At conception (1976–1983)					
All mothers	11.8	16.2	26.0	58.9	232.3
Mothers who breast-fed^b^	11.8	13.1	19.0	58.9	117.1
Mothers who formula-fed^c^	17.0	20.6	27.9	54.6	240.3

Mothers of comparisons were considered as exposed to average background TCDD level of 10 ppt in 1976 (Eskenazi et al. 2004).

a Values are parts per trillion TCDD concentration expressed on serum lipid basis.

b After breast-feeding for 4–5 months, the TCDD value in the child roughly doubles compared with the conception value (Abraham et al. 1998).

c The formula-fed men were exposed to dioxin only *in utero*.

**Table 3:** Differences in sperm and hormone data between men exposed to dioxin *in utero* (i.e., formula-fed) or also through breast-feeding and same-age comparisons.

			Breast-fed		Formula-fed		*p*-Value,^a^ breast-fed versus formula-fed	
Characteristic	Exposed group (*n* = 39)	Comparison group (*n* = 58)	Exposed group (*n* = 21)	Comparison group (*n* = 36)	Exposed group (*n* = 18)	Comparison group (*n* = 22)	Exposed group	Comparison group
Semen volume (mL)								
Median (25th–75th percentile)	3.0 (2.2–3.8)	3.3 (2.4–4.3)	3.0 (2.3–3.8)	3.4 (2.1–4.2)	3.0 (2.0–3.8)	3.0 (2.4–4.4)		
Adjusted mean^b^ (−/+ SE)^c^	3.2 (3.0/3.4)	3.0 (2.8/3.2)	3.4 (3.1/3.7)	2.9 (2.6/3.1)	3.0 (2.7/3.3)	3.2 (2.9/3.5)		
*p*-Value^a^	0.60		0.21		0.68		0.39	0.38

Sperm concentration (10^6^/mL)								
Median (25th–75th percentile)	61.5 (32.1–100.0)	88.7 (54.6–133.1)	54.4 (22.1–68.5)	99.6 (58.2–141.6)	81.1 (37.2–107.3)	81.8 (41.4–96.1)		
Adjusted mean^b^ (−/+ SE)^c^	46.2 (39.8/53.6)	81.0 (71.9/91.1)	36.3 (29.8/44.2)	86.3 (74.1/100.6)	60.8 (49.4/74.9)	73.2 (60.5/88.6)		
*p*-Value	0.01		0.002		0.53		0.07	0.51

Sperm total count (10^6^)								
Median (25th–75th percentile)	143.9 (78.1–336.2)	230.6 (162.6–422.3)	108.2 (57.0–209.0)	270.8 (178.1–431.8)	222.0 (118.3–349.2)	214.1 (134.4–359.7)		
Adjusted mean^b^ (−/+ SE)^c^	139.2 (119.3/162.4)	229.9 (203.4/259.9)	116.9 (95.1/143.6)	231.1 (197.0/271.2)	171.4 (137.9/213.1)	227.0 (185.9/277.1)		
*p*-Value	0.03		0.02		0.36		0.19	0.94

Sperm progressive motility (%)^d^								
Median (25th–75th percentile)	39.0 (33.0–47.0)	41.5 (34.0–50.0)	37.0 (31.0–44.0)	43.5 (38.5–51.5)	40.0 (37.0–48.0)	36.5 (32.0–49.0)		
Adjusted mean^b^ (−/+ SE)^c^	38.7 (36.3/40.9)	41.5 (39.8/43.2)	35.8 (33.0/38.5)	44.2 (42.0/46.3)	41.7 (38.8/44.6)	37.4 (34.8/40.1)		
*p*-Value	0.47		0.03		0.30		0.13	0.06

Progressive motile sperm count (10^6^)^e^								
Median (25th–75th percentile)	58.5 (24.8–141.2)	98.1 (65.9–193.9)	44.4 (21.1–81.2)	111.7 (75.0–197.1)	87.2 (42.6–143.2)	94.9 (41.7–150.2)		
Adjusted mean^b^ (−/+ SE)^c^	50.6 (41.4/61.8)	90.5 (77.1/106.2)	38.7 (29.6/50.6)	98.0 (79.7/120.6)	68.5 (51.6/90.8)	80.0 (61.7/103.6)		
*p*-Value	0.05		0.01		0.70		0.13	0.55

FSH (IU/L)								
Median (25th–75th percentile)	3.2 (2.1–5.3)	2.7 (1.9–4.7)	3.7 (2.9–6.4)	2.6 (1.8–4.7)	2.7 (1.6–3.8)	3.2 (2.3–4.7)		
Adjusted mean^b^ (−/+ SE)^c^	3.3 (3.0/3.7)	2.7 (2.4/2.9)	4.1 (3.5/4.8)	2.6 (2.3/3.0)	2.6 (2.2/3.1)	2.8 (2.4/3.2)		
*p*-Value	0.23		0.03		0.79		0.04	0.79

Inhibin B (pg/mL)								
Median (25th–75th percentile)	77.6 (49.3–114.7)	99.6 (86.4–129.7)	73.2 (49.3–89.9)	105.7 (87.6–126.7)	87.0 (50.2–133.2)	96.0 (83.4–133.2)		
Adjusted mean^b^ (−/+ SE)^c^	83.2 (76.4/90.3)	100.0 (94.0/106.2)	70.2 (62.0/78.9)	101.8 (94.0/109.8)	99.9 (89.5/110.8)	97.2 (87.8/107.0)		
*p*-Value	0.13		0.01		0.85		0.02	0.72

a *p*-Values refer to differences adjusted by abstinence time (days), BMI, smoking status and total number of cigarettes per day during months of habitual smoking, age at the time of test, chemical substances exposure, alcohol use (g/day), and education level for sperm data. Hormone data were not adjusted for education level, employment status, and abstinence time.

b Values derived from back-transformation of log (sperm concentration, sperm total count, progressive motile sperm count, and FSH) and square-root transformation (semen volume and inhibin B).

c Values indicate adjusted mean minus the SE/adjusted mean plus the SE.

d Considered as A + B grades of progressive motility of sperm, according to WHO (1999).

e Considered as A + B grades of progressive motility of sperm per total sperm count, according to WHO (1999).
